# Transcription factor site dependencies in human, mouse and rat genomes

**DOI:** 10.1186/1471-2105-10-339

**Published:** 2009-10-16

**Authors:** Andrija Tomovic, Michael Stadler, Edward J Oakeley

**Affiliations:** 1 Friedrich Miescher Institute for Biomedical Research, Novartis Research Foundation, Basel, Switzerland; 2 Modeling and Simulation, Novartis Pharma AG, Basel, Switzerland

## Abstract

**Background:**

It is known that transcription factors frequently act together to regulate gene expression in eukaryotes. In this paper we describe a computational analysis of transcription factor site dependencies in human, mouse and rat genomes.

**Results:**

Our approach for quantifying tendencies of transcription factor binding sites to co-occur is based on a binding site scoring function which incorporates dependencies between positions, the use of information about the structural class of each transcription factor (major/minor groove binder), and also considered the possible implications of varying GC content of the sequences. Significant tendencies (dependencies) have been detected by non-parametric statistical methodology (permutation tests). Evaluation of obtained results has been performed in several ways: reports from literature (many of the significant dependencies between transcription factors have previously been confirmed experimentally); dependencies between transcription factors are not biased due to similarities in their DNA-binding sites; the number of dependent transcription factors that belong to the same functional and structural class is significantly higher than would be expected by chance; supporting evidence from GO clustering of targeting genes. Based on dependencies between two transcription factor binding sites (second-order dependencies), it is possible to construct higher-order dependencies (networks). Moreover results about transcription factor binding sites dependencies can be used for prediction of groups of dependent transcription factors on a given promoter sequence. Our results, as well as a scanning tool for predicting groups of dependent transcription factors binding sites are available on the Internet.

**Conclusion:**

We show that the computational analysis of transcription factor site dependencies is a valuable complement to experimental approaches for discovering transcription regulatory interactions and networks. Scanning promoter sequences with dependent groups of transcription factor binding sites improve the quality of transcription factor predictions.

## Background

Transcription factors (TFs) are a major class of DNA-binding proteins and are a crucial element in the regulation of gene expression. It is well established that many transcription factors act together to regulate gene expression in eukaryotes [1]. For example, the cooperation between E2F and NF-Y, two main regulators of cell cycle, has been described in [2,3]. A commonly used experimental method to identify interacting proteins is tandem affinity purification (TAP), as reviewed in [4]. This approach requires the expression of recombinant fusion proteins, which is laborious, may interfere with protein function and may lead to non-physiological expression levels of the studied protein. A computational detection of potential interacting transcription factors could therefore complement experimental approaches. There are many prediction tools and databases of composite motifs and cis-regulatory modules (multiple transcription factor binding sites in a strict order and spacing) [5-21]. Most of the tools for predicting cis-regulatory modules have been limited by rigid assumptions on the architecture of the module, such as length, number and order of contained cis-motifs, distance between cis-motifs, and the DNA strand on which a binding site must appear. It has been shown that 98% of 375 known vertebrate composite elements have a distance of less than 100 bp [22]. Although these assumptions can be valid for the detection of cis-regulatory modules, they are too restrictive to allow sensitive detection of binding sites dependencies. Transcription factor cooperativity can be achieved with different spatial arrangements on different promoters. There are, for example, transcription factors which co-occur and bind to the promoter at very large distances (>1 Kbp) between them (such as GAGA and Gal4 [23]). In order to overcome these restrictions, we investigated transcription factor binding sites dependencies in terms of how often their predicted binding sites are found together within a window extending 1.5 Kb 5' and 200 bp 3' of the putative starts of transcription in human, mouse and rat genes, without any further assumption on their binding characteristics. This leads to an approach that differs from prior approaches for detecting cis-regulatory modules. Dependencies between transcription factor binding sites are evaluated using only co-occurrences among different promoter sequences, disregarding any information on arrangement and counts of occurrences within the same promoter. Binding sites of two transcription factors that appear significantly more often together (among different promoters) than expected are indicative of a dependency between them. Using this approach, even dependencies between sites that do not occur in a strictly defined order and spatial organization can be identified. Our approach for quantifying tendencies of transcription factor binding sites to co-occur is based on a scoring function which incorporates dependencies between nucleotides [24], the use of information about the structural class of each transcription factors (minor or major groove binder) and considering the possible implications of varying GC content of the sequences. The significant tendencies (dependencies) have been detected by non-parametric statistical methodology (permutation tests). Evaluation of obtained results has been performed in several ways: reports from literature (many of the significant dependencies between transcription factors have previously been confirmed experimentally); dependencies between transcription factors are not biased due to similarities in their DNA-binding sites; the number of dependent transcription factors that belong to the same functional and structural class is significantly higher than would be expected by chance; supporting evidence from GO clustering of targeting genes. The only restriction our method applies is to limit the search to the 1.7 Kb window described above, without any further restrictions on the distance between or the organization of the binding sites (cis-motifs).

Based on dependencies between two transcription factor binding sites (second-order dependencies), it is possible to construct higher-order dependencies (networks). Obtained results about dependencies among transcription factor binding sites have been further used for development of a web-based tool that allows scanning of promoter sequences for groups of dependent transcription factor binding sites . This tool can help in predicting transcription factor binding sites in promoter analysis with relatively high sensitivity and modest specificity (which is still higher in comparison to single site prediction tools (such as [24]).

## Results and Discussion

### Distributions of dependencies between transcription factors

From the JASPAR database, we selected all vertebrate transcription factors (August 2007, total: 76) and made all the possible 2-order combinations (in total:  = 2850). There is no comprehensive transcription factor database that would list all transcription factors with their target binding sites. From publicly available databases, JASPAR is currently the best annotated transcription factor database (new version of JASPAR database has appeared in 2008 with 88 vertebrate transcription factors). Using promoter sequences of all human, mouse and rat annotated genes (see Materials and Methods section), we analysed transcription factor site dependencies (see Material and Methods section). The total number of significant dependencies (significance level of 0.05/k, k = 75, see Methods section) in the human, mouse and rat genomes were 1438 (50.5%), 1239 (43.5%) and 1063 (37.3%), respectively [see Additional file 1]. The corresponding numbers of significant dependencies observed on background sequences [see Additional file 1] are significantly smaller (Fisher's exact test, p-value < 0.001), and are about as high as expected based on the p-value threshold (0.05*2850). On average, the numbers of significant dependencies observed in the human, mouse and rat genomes are about four times higher than those found in the background sequences, which may indicate that statistical dependencies could correspond to real biological dependencies between transcription factors. The number of the common dependent pairs between species was also analysed [see Additional file 1] and we found a high conservation between species in terms of transcription factor dependencies, further supporting the validity of our results. Additional supporting evidence for our findings was found from the literature for many of the significant transcription factor combinations [25-29]. For example, it has been reported that SP-1 and E2F interact directly in delivering an activation signal to the basic transcription machinery [25]. In our computational analysis, dependencies between binding sites of SP-1 and E2F were detected separately in human, mouse and rat genomes, with p-values < 0.0001 in each case. There was a similar situation for USF1 and RUNX1: dependency was predicted in all three genomes, with p-values < 0.0001, and it has been reported that they interact with each other [26]. Another example is the MAX and MYC-MAX dependency which, as well as the MAX and MYCN dependency, was predicted in all three genomes, with a p-value < 0.0001, and has previously been identified [27]. The MAX-USF, MYC-USF dependency (p < 0.0001) was described in [28], NFkappaB-RELA, NFKB1-REL (p < 0.0001) in [29], and the E2F1-NFY dependency (p < 0.0001) in [2,3]. There are many other confirmatory examples which agree with the computationally predicted transcription factor dependencies. However, in order to perform a detailed investigation of the number of true and false positives we would need a precise text-mining tool to search the available scientific literature. Moreover, an additional limitation for such an investigation is that experimental information available in the literature about interacting transcription factors is certainly incomplete. Because of this, some of the results that have been evaluated as incorrect predictions (false positives) may in fact be true positives.

For each transcription factor, we analyzed the number of its dependent mates in human, mouse and rat genomes. The distributions of dependent mate numbers [see Additional file 2] are very heavily skewed from Gaussian (significantly different from Normal distributions with p-value < 0.01 detected by Kolmogorov-Smirnov, Cramer-von Mises or Anderson-Darling test for all 3 genomes) and follow a U-shaped distribution (e.g. Beta(a, b), a<1,b<1). That was expected according to the fact that there are "popular" (very often seen in dependent pairs) and "unpopular" (rarely seen in dependent pairs) transcription factors. For example a popular transcription factor in all three genomes is CREB. CREB was found to regulate ~4000 target genes in the human genome, and a majority of these are occupied in vivo [30]. In addition, there is a large number of CREB-occupied loci in the rat genome [31].

Some transcription factors, such as GATA2 and EN1, have a very high number of predicted binding sites and are thus predicted to regulate a large fraction of the analyzed promoters. For such factors, a higher number of co-occurrences with other binding sites can be observed. While our statistical approach will take this into account through an increased number of expected random co-occurrences, we wondered whether this could still cause a bias in our results. We have therefore performed a correlation analysis between the number of predicted single binding sites and the number of dependent mates for each transcription factors. We used the "Significance test for Pearson correlation" which is valid for sample sizes where N > 6 to assess these correlations. The Pearson's correlation coefficients were 0.04 (p-value = 0.75), -0.27 (p-value = 0.02) and -0.39 (p-value < 0.01) for human, rat and mouse, respectively. These results indicate that there might be reduced statistical power for factors with many predicted sites (correlation coefficient significantly different from zero in the case of rat and mouse), potentially because their lower site information content could give rise to more noise in the site predictions. However, weak correlation coefficients imply small influence of such noise on obtained results.

Similarly, we investigated the influence of binding site length on the number of dependent mates. Short binding sequences could increase the frequency of detected binding sites. We have therefore performed a correlation analysis between the length of binding sites and the number of dependent mates for each transcription factor. The Pearson's correlation coefficients were -0.30 (p-value < 0.01), -0.17 (p-value = 0.14) and -0.06 (p-value = 0.60) for human, rat and mouse, respectively. These results indicate that at least for the analysis in human, shorter binding sites tend to give rise to more dependent pairs. We cannot rule out that this is due to a higher number of false positive predictions associated to TFs with short binding sites. Yet, the observed correlation coefficients are weak, and for mouse and rat not significantly different from zero. This indicates that the resulting bias is weak and does not dominate our results.

Another potential source of bias could be the sequence composition of the promoters and binding motifs. For example, a GC-rich promoter sequence would be more likely to contain predicted sites for GC-rich binding motifs, and detection of dependencies between corresponding factors could be biased. The stratification according to GC-content used by our resampling approach should control for the GC-content, but other compositional biases might exist that we did not account for. To investigate this issue, we performed a clustering of transcription factors based on the similarity between their binding sites [see Additional file 3]. This kind of clustering is performed in [32], and we observed [see Additional file 3] that only few TFs had sufficiently similar binding site specificities to be grouped together: the top two clusters are Cluster-15 (containing 6 transcription factors) and Cluster-5 (containing 5 transcription factors). The other clusters contain less than 5 TFs, and 32 clusters only contain a single TF. Moreover, the most popular/unpopular transcription factors (we define a popular TF as a TF which is involved in many pairwise interactions) always belong to different clusters (do not have similar binding sites), with only one exception with two popular transcription factors (ARNT, USF1). We then analyzed if dependent pairs are more likely to belong to the same cluster (Table 1). In 25 out of 469 dependent pairs (5.3%), both transcription factors are part of the same cluster. Over all possible transcription factor pairs, both factors belong to the same cluster in 33 of 1507 pairs (2.2%). This indicates that similar binding site specificity might increase the chance to be dependent by about 2.5-fold, but would still only account for a minority of predicted dependent pairs. Taken together, these results suggest that dependencies between transcription factors cannot be explained by similarity of their DNA-binding sites.

**Table 1:** Distributions of pair dependencies according the binding sites similarity clustering.

	Dependent pairs A-B*	Independent pairs A-B*
**A&B belong to the same cluster**	25	8
**A&B belong to the different cluster**	444	1063

p-value = 7.692106e-08 (Fisher's exact test)

*transcription factors for which cluster is not assigned [see Additional file 3] are omitted from analysis

The number (percent) of dependent/independent pairs (in all there genomes human+mouse+rat intersection) that belong to the same/different cluster (clustering of transcription factors is performed based on the similarity between their binding sites, see Additional file 3).

**Figure 1:**
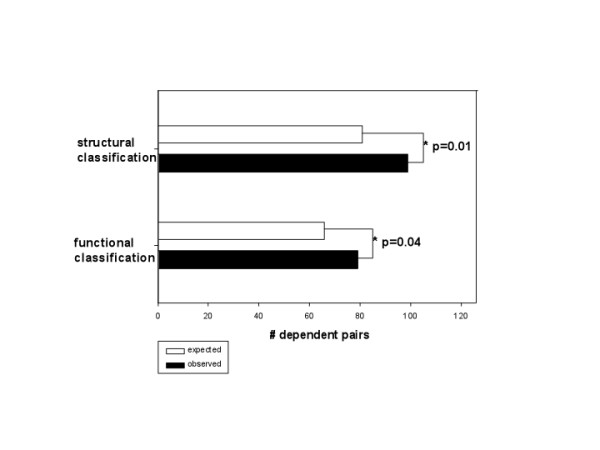
**Distributions according to the structural and functional classification**. Expected (random) and observed distributions of dependent pairs of TFs which belong to the same structural/functional class (* p < 0.05, Chi-square test; Expected distribution gives the numbers of dependent pairs of transcription factors which belong to the same structural/functional class that one would expect to obtain if there is no difference between proportions of dependent pairs that contain transcription factors from the same and different structural/functional classes).

Next, we investigated how many dependent pairs contain transcription factors that belong to the same structural class, using the classification from JASPAR [33]. It has been reported that transcription factors from the same structural class tend to bind in a similar way [33-37]. We found that belonging to the same structural class is related to dependencies between transcription factors (Figure 1). This is also in agreement with the statement that similar structures imply similar functions, and similar functions imply possible transcription factor binding site dependencies. An alternative way of classifying transcription factors is based on their functions (i.e. biological processes) obtained from [38]. We investigated the distribution of dependencies according to this classification (which only covers 51 of the 76 factors used in this work), in a similar way to the structural classification. In this situation (which is more relevant for this study), we expected that transcription factors that belong to the same functional group (have the same or similar biological processes) should be dependent more often than transcription factors from the different functional class. Indeed, the number of dependent transcription factors that belong to the same functional class is significantly higher (p = 0.04, Chi-square test) than randomly expected in the human, rat and mouse genomes (Figure 1). For the functional analysis we did not use the all transcription factors used in this study, because for some there was no reported functional class available in [38]. This could have limited our statistical sensitivity and might be the reason why the functional enrichment was only marginally significant.

**Figure 2:**
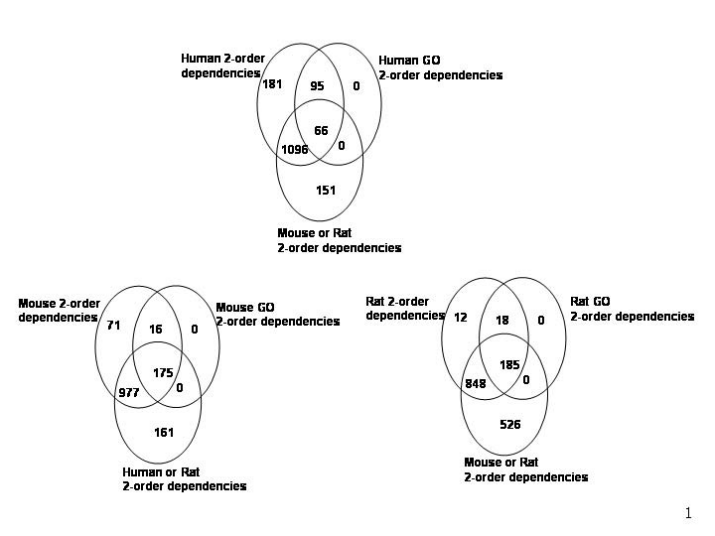
**Venn diagrams of the number of dependent transcription factor binding sites pairs in human, mouse and rat genome**. Venn diagrams show the number of total predicted dependent pairs, the number of predicted dependent pairs conserved in two or three species, the number of predicted dependent pairs supported by GO, and the number of predicted dependent pairs supported by overlapped supporting evidence.

Finding groups of genes that are correlated throughout a set of experiments leads to the hypothesis that these genes are involved in common functions [39]. Further, we can expect that these genes have similar sets of dependent transcription factor binding sites. Knowledge of these sets may be crucial for further understanding of regulatory networks. Following this we investigated distributions of dependent transcription factor binding sites using the GO ontology classification (biological process and molecular function) of target genes whose promoters we used in the study, using only GO classes that contained at least 25 genes. Clustering of dependent TFs was performed in the following way: each dependent pair of TFs which had in its target list at least 80% of promoters (genes) that belong to the given GO class is assigned as relevant for that class. All results are available from . The predictions of dependent transcription factor binding sites are more likely to be true if they are supported by multiple lines of evidence. Figure 2 represents Venn diagrams for human, mouse and rat results separately. Venn diagrams show the number of total predicted dependent pairs, the number of predicted dependent pairs conserved in two or three species, the number of predicted dependent pairs supported by GO, and the number of predicted dependent pairs supported by overlapped supporting evidence. We can see that the highest number of dependent pairs is supported by 2 evidences and all dependent pairs from GO analysis are supported by other 2 evidences for each species, further supporting the validity of our results. Another potential way to investigate dependencies between transcription factors according to the GO classification of their target genes would be to group the promoters belonging to the same GO cluster and perform the same analysis (see section 2.1) as performed previously with the set of all promoters. However, in practice this approach proved under-powered because of the limited number of promoters in each GO class. There were too few promoters to apply the same re-sampling techniques used for the whole genome.

It is likely that some protein-DNA complexes not only contain two, but three or more cooperating transcription factors. In order to identify such groups of more than two dependent sites, one could apply the same method as for pairs. In practise however, it is not feasible to enumerate and analyze all combinations of three or more transcription factor binding sites (for example, there are 70300 groups of three and over 1.2 million groups of 4 factors from Jaspar). Instead, we used the results on significantly associated pairs for extrapolation. Starting from dependencies of order two, we analyzed the dependencies of higher orders as fully or partially connected transcription factor networks. To make all results easily accessible, we have provided a web-based tool, freely accessible from  where users can search by transcription factor name and retrieve our results on dependencies (full and partial). For stringent searching, users can require the transcription factor network to be fully connected (e.g. for A-B-C dependencies it is necessary to have A-B, A-C and B-C dependencies) and represents exactly the results which would be obtained via direct enumeration. Partial connectivity is less stringent (e.g. for third-order only two combinations are necessary to be dependent) and represents a less stringent approximation of the full enumeration results. Information obtained in this way can be useful for designing biological experiments where information about transcription factors that may cooperate is useful (design of regulatory gene networks for various processes). In addition, the results obtained about dependencies are potentially useful for better understanding transcriptional networks in human, mouse and rat genomes.

### Computational prediction of groups of dependent transcription factors binding sites

Results from descriptive data-mining about dependencies between transcription factor binding sites can be used for the computational prediction of modules of dependent binding sites. In order to evaluate the proposed tool, we used experimentally verified data from [40,41]. From the dataset of transcription factors which we used in this study, we selected a subset which was known to be involved in the regulation of skeletal muscle gene expression: MEF2, SP-1, SRF, MZF1_1-4 and MZF1_5-13. It is known that a set of nine human genes (NM_184041, NM_001927, NM_002479, NM_079422, NM_003281, NM_000257, NM_002471, NM_001100 and NM_005159) is regulated by combinatorial interactions between the transcription factors listed above [41]. First, we noticed that based on second order dependencies (in human) among these transcription factors (Table 2) it was possible to construct fifth-order partial dependencies between them. We used the 2 Kbp upstream region of the nine human genes and scanned them with modules of order 2. We found (Table 2) that almost all second-order modules were detected in all nine promoters.

**Table 2:** Computational prediction of groups of dependent transcription factors binding sites.

**2-order TF dependency**	**# (%)of promoters where module has been detected**
MZF1_5-13 ↔ SP-1	9 (100%)

MZF1_1-4 ↔ MZF1_5-13	9 (100%)

MZF_1-4 ↔ SP-1	9 (100%)

MEF2 ↔ SRF	1 (11%)

General form of output after scanning promoter sequences for the given combination of transcription factors A and B.

Only module MEF2-SRF was not detected in all sequences, however there are other combinations that include one of these two transcription factors detected in more sequences. This is not a surprise because not only these 5 transcription factors are involved in the regulation of skeletal muscle genes.

In order to further demonstrate the practical application of the proposed tool, we can simulate the following scenario: if we know that one specific transcription factor is involved in the regulation of a set of genes, and we would like to know which other possible transcription factors might be involved, then we could use the proposed tool to create a list of candidates. Specifically, using the set of nine genes that showed skeletal muscle expression we could start from the any of the 5 mentioned transcription factors and then find the factors that might interact with it in the regulation of these nine genes. Using the proposed tool, we were able to predict all the other known transcription factors reported to be involved in the regulation of these genes (true positives). However, we also determined another set of transcription factors for which no experimental support exists (which we might consider as potential false positives).

In order to perform more detailed validation test, we used transcription factors that were predicted and experimentally identified as true positives, transcription factors that were not predicted but experimentally reported for a given promoter as false negatives, transcription factors that were neither predicted nor experimentally reported as true negatives and transcription factors that are predicted but not experimentally reported are false positives (Table 3, with muscle specific data from [40,41]). The second order dependencies have been used in this evaluation. In addition, we have used promoters (and corresponding transcription factors: HLF, TCF1(HNF1), FOXa2 (HNF3), RORA, SOX17, cEBP, HNF4) of human liver specific genes from [42] and performed similar validation (Table 4). We noticed that sensitivity is relatively high and specificity relatively low. While our method could detect almost all true positives from both experiments, it produced many false positive predictions similar to other tools for prediction of transcription factor-binding sites. However, it is important to mention that it is not guaranteed that the experimentally reported transcription factors represent the complete set of factors for the given genes (true positives). Therefore, some of the false positives might be true positives and the actual specificity could be higher than estimated here. In comparison to single site prediction tools (such as [24], Table Sup eight-three and tools reported there), our tool has an increased specificity and sensitivity.

**Table 3:** Evaluation of prediction of dependent transcription factor binding sites using transcription factors involved in the regulation of skeletal muscle gene expression.

**Promoter of human gene (Gene RefSq ID)**	**TP**	**TN**	**FP**	**FN**	**Specificity**	**Sensitivity**
NM_000257	3	21	50	2	0.32	0.6

NM_001100	4	24	47	1	0.35	0.8

NM_001927	4	25	46	1	0.36	0.8

NM_002471	3	25	46	2	0.37	0.6

NM_002479	4	20	51	1	0.29	0.8

NM_003281	4	22	49	1	0.32	0.8

NM_005159	5	22	49	0	0.31	1

NM_079422	4	21	50	1	0.31	0.8

NM_184041	3	27	45	1	0.38	0.75

TP-true positives, FP-false positives, TN-true negative, FN-false negative, sensitivity = TP/(TP+FN), specificity = TN/(TN+FP)

**Table 4:** Evaluation of prediction of dependent transcription factor binding sites using transcription factors involved in the regulation of human liver.

**Promoter of human gene (Ensembl ID)**	**TP**	**TN**	**FP**	**FN**	**Specificity**	**Sensitivity**
ENSG00000150526	6	23	46	1	0.33	0.857

ENSG00000017427	6	20	49	1	0.29	0.857

ENSG00000084674	6	23	46	1	0.33	0.857

ENSG00000115718	5	23	46	2	0.33	0.714

ENSG00000116833	6	28	41	1	0.41	0.857

ENSG00000126218	6	21	48	1	0.30	0.857

ENSG00000136872	6	20	49	1	0.29	0.857

ENSG00000163581	6	25	44	1	0.36	0.857

ENSG00000163631	6	21	48	1	0.30	0.857

ENSG00000167165	6	28	41	1	0.40	0.857

ENSG00000167910	6	27	42	1	0.39	0.857

ENSG00000171759	6	26	43	1	0.37	0.857

ENSG00000173531	6	23	46	1	0.33	0.857

ENSG00000180432	6	23	46	1	0.33	0.857

ENSG00000101076	6	22	47	1	0.32	0.857

ENSG00000163631	6	21	48	1	0.30	0.857

ENSG00000145321	6	23	46	1	0.33	0.857

ENSG00000169562	6	22	47	1	0.32	0.857

ENSG00000132437	6	21	48	1	0.30	0.857

ENSG00000105398	6	24	45	1	0.35	0.857

ENSG00000131482	6	25	44	1	0.36	0.857

ENSG00000198610	6	25	44	1	0.36	0.857

TP-true positives, FP-false positives, TN-true negative, FN-false negative, sensitivity = TP/(TP+FN), specificity = TN/(TN+FP)

## Conclusion

In this paper we describe a data-mining study to identify transcription factor site dependencies in the human, mouse and rat genomes. Many of the predicted dependent transcription factors had been confirmed previously *in vitro *or *in vivo *and have been reported in the literature: these represent partial validation of our approach (agreement between statistical and biological/experimentally confirmed/dependencies). Dependencies between transcription factors are not biased by similarities in their DNA-binding sites. The distribution of transcription factors, whose binding sites are dependent, according to their functional classification shows that they tend to be involved in same biological process. Genes that are involved in common functions tend to have similar sets of dependent transcription factor binding sites. Knowing these sets may further our understanding of gene regulation networks. This is why we provided distributions of dependent transcription factor binding sites in GO ontology classes of target genes whose promoters we used in the study and these results are available from . Starting from the dependencies of order 2, it is possible to construct higher order dependencies (networks). All results can be obtained via the web tool . This information may help others in their investigation of transcriptional processes in human, mouse and rat. In addition, we demonstrated how the information obtained about dependencies could be used for the computational prediction of modules of dependent transcription factor binding sites . We validated the tool using experimentally verified data set of transcription factors involved in the regulation of skeletal muscle expression. We also demonstrated how the proposed tool might be applied. Computational analysis of transcription factor site dependencies is a complement to experimental approaches for discovering transcription regulatory interactions and networks.

## Methods

### *De novo *detection of transcription factor site dependencies

The dataset used in this study comprised promoter sequences (1500 bp upstream to 200 bp downstream of annotated transcription start sites) of 18,799 human (Ensembl Build 40, NBCI v36, hg18), 17,954 mouse (Ensembl v38, NCBI m35, mm7) and 6,723 rat genes (Ensembl v22, NCBI v3.1, rn3) taken from the cisRED database, August 2007 [43]. The set of vertebrate transcription factors (total 76) with their binding sites was obtained from the non-redundant, curated and publically available database JASPAR [44,45] (August, 2007). We also used negative control sequences as a background in order to see how many dependent transcription factors can be found in sequences which are not real promoters of selected genes. Background sequences were generated for each species as described in [43], of 1000 concatenated search regions that were randomly selected from the genome's entire set of search regions.

In order to detect transcription factor site dependencies, we first enumerated all second-order combinations of transcription factors. Then, using the new scoring function introduced in our previous work [24], we predicted binding sites for the given combination of transcription factors on the aforementioned human/mouse/rat promoter sequences. It is difficult to define a single optimal score threshold for all TFs. Individually optimized thresholds might be necessary to account for varying degrees of specificity inherent to some TFs. Nevertheless, we used universal but distance specific thresholds for this study: 0.88 if the distance between binding sites was longer than 5 bp, otherwise 0.80, because transcription factors with direct contacts between them can make more stable complexes with DNA even though their DNA-binding affinities may be lower, as discussed in [46]. In our previous paper [24] we suggested values between 0.8 and 0.9 as optimal medium stringency thresholds for the prediction of single transcription factor binding sites. Very similar results are obtained if other thresholds are chosen from this interval, with a ~5-10% difference between them (data not shown). In addition for detection binding site dependencies, we also included information about the structural class of each transcription factor from the JASPAR database. It is known that most transcription factors bind to the major DNA groove, but some of them bind to the minor groove. Practically, this means that overlapping binding sites can be possible if one transcription factor binds to the major and other to the minor groove (acceptable structural arrangement). The strand of DNA determines the orientation of transcription factors on DNA. Based on this observation, we allow that the binding sites of two transcription factors can overlap (partially or even completely) if those two transcription factors bind to DNA in a different way (one to the major and one to the minor groove). We analyzed both strands of the promoter sequences. In summary, if there are two binding sites (of different transcription factors) are further apart than 5 bp, we treated them as "predicted" if scoring function is higher than 0.88. If the distance is shorter then 5 bp (or there is overlap between them) with acceptable structural arrangement we treated them both as "predicted" even if scoring function for any of them is smaller of 0.88 (but >= 0.8); finally if two binding sites (of different transcription factors) overlap with an unacceptable structural arrangement, then we treated only the one with the higher score as "predicted".

For each promoter sequence we calculated the CG context (%G + %C). Histogram distributions of GC content are given in Additional file 4. We employed a Monte-Carlo re-sampling approach to determine the significance of observed co-occurring transcription factor binding sites as follows. For a given combination of two transcription factors A and B, and the list of promoter sequences, the results of the initial predictions can be represented as a table in which we have calculated the number of promoter sequences that have binding sites for both transcription factors A and B [see Additional file 5]:

(1)

where

(2)

and n is the total number of sequences, A_i _= 1 means that sequence i has binding sites of transcription factor A, A_i _= 0 means that sequence i has no binding sites of transcription factor A, and similar for B_i_.

Then, in a series of R replicates, we performed a permutation of the initial table [see Additional file 5] in the following way: for each promoter sequence i (1 ≤ i ≤ n), we randomly assigned to it another promoter sequence j (1 ≤ j ≤ n) which had a similar GC content, and we replaced (swapped) values in column A between rows (sequences) i and j (i.e. A_i _<-> B_j_).

In order to define the term "similar GC content between sequences" we could have used equal intervals of GC content. However, we noticed that this would result in a smaller number of sequences for permutation in high and low GC bins. To correct for this, we produced 50 bins with a fixed number of promoters per bin [see Additional file 6]. In this way, we ensured enough possible permutations for each sequence and its corresponding GC content. Using this method, we produced R permuted tables, and for each permuted table we counted how many times we had the value 1 in columns A and B (CountPermjAB was performed substituting "CountPermAB" for "CountAB" in equation (1)) for each table j (j = 1,... R). Finally, a p-value was calculated in the following way:

(3)

where

(4)

and R is the resample size (number of replicates), and adding 1 is the pseudocount that prevents us from underestimating the p-value when it is low or zero. We used an adjusted p-value (with Bonferroni's correction) to correct for multiple testing errors. Dependencies were declared significant if the computed p-value was smaller than 0.05/k (where k is the number of multiple tests). We determine the number of re-sampling runs using the following formula:

(5)

where P_threshold is the significance p-value threshold selected which, in our case, corresponded to P_threshold = 0.05/k where k = 75. We therefore selected R = 15,000 as a compromise between accuracy in p-value estimation and calculation time (R>>k/0.05 = 1500).

### Higher-order transcription factor site dependencies

Starting from dependencies of order two, we constructed dependencies of higher orders in the following way: if transcription factors A-B, B-C and A-C are all dependent, then we can claim that there is an order three dependency between transcription factors A, B and C. (Note: it is not true if only A-B and B-C are dependent pairs but A-C is not). Third-order dependencies between the transcription factors A, B and C can be represented as fully connected graph as shown in Additional file 7. Other forms of third-order dependencies (partial third-order dependencies) of transcription factors (when any of two pairs of three transcription factors are dependent) can be represented using a not fully connected graph [see Additional file 7]. Higher order dependencies between factors can be represented in a similar way.

### Scanning tool for predicting groups of dependent transcription factor binding sites

The computational prediction of cis regulatory motifs of dependent transcription factors in scanning form can be performed using information about dependencies between transcription factor binding sites using the scoring function which we introduced in a previous paper [24] and, in addition, structural information (possible position binding) between transcription factors as we described in section "De novo detection of transcription factor site dependencies". We used universal but distance specific thresholds for the scoring function as described in the same section. This method is implemented as a web-based tool and it is available from: . Different cut-off values in the range between 0.8 and 0.9 only had a minor influence on the results in Table 3 and 4 (slightly varying only in the number of false positives for different promoters from the here shown numbers for different cut-off values). If very different cut-off values are chosen (above 0.9 or bellow 0.8), a greater impact on the results as shown in Table 3 and 4 can be observed. As indicated in the section "De novo detection of transcription factor site dependencies", we think however that it is not recommended to use such cut-off values.

## Authors' contributions

AT designed the study, performed computational analysis, created supported web tool and drafted the manuscript. MS and EJO participated in the design of the study, discussion of the results and drafting of the manuscript. All authors read and approved the final manuscript.

## Supplementary Material

Additional file 1 **Distribution of dependencies of order 2 in the human, mouse and rat genomes using real promoters sequences and background sequences.** Click here for file

Additional file 2 **Distributions of number of dependent mates in human, mouse and rat genome**. File containing 3 histograms of number of dependent mates for each transcription factor in human, mouse and rat genome. Click here for file

Additional file 3 **Distribution of dependent mates for each transcription factor in human, mouse and rat genome, including cluster information about similarity between binding sites.** Click here for file

Additional file 4 **Distribution of GC content in the human, mouse and rat promoters**. File containing 3 histograms and corresponding fitted normal distributions. Click here for file

Additional file 5 **Scanning promoter sequences**. File containing a table that represents a general form of output after scanning promoter sequences for the given combination of transcription factors A and B. Click here for file

Additional file 6 **Distributions of GC content in human promoters, represented by a histogram of 50 bins**. File containing 3 histograms of 50 bins each. Click here for file

Additional file 7 **Representation of higher order dependencies between transcription factors A, B and C**. File containing fully connected graph (represents full 3-order dependencies) and not fully connected graph (represents partial 3-order dependencies). Click here for file
