# Supplementary material for: Transcription factor site dependencies in human, mouse and rat genomes
Source: BMC Bioinformatics. 2009 Oct 16;10:339. doi: 10.1186/1471-2105-10-339 (PMC2770556; doi:10.1186/1471-2105-10-339)
Supplement: Additional file 1 — Distribution of dependencies of order 2 in the human, mouse and rat genomes using real promoters sequences and background sequences. [file 1471-2105-10-339-S1.PDF]

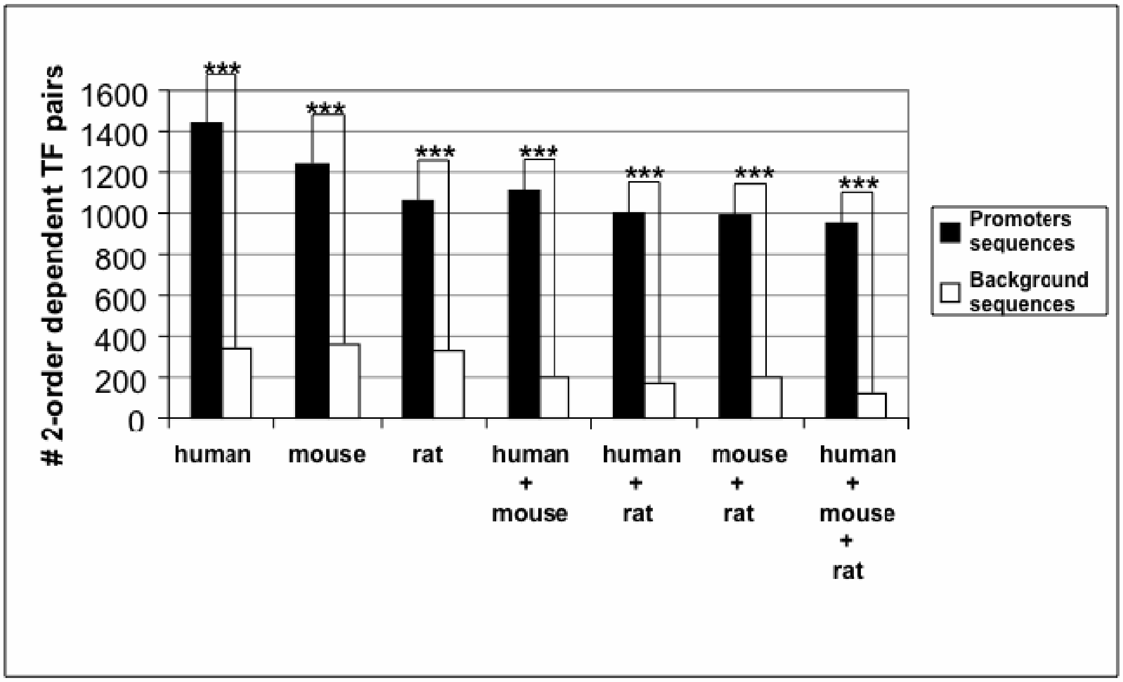

Dependency distributions

Distribution of dependencies of order 2 in the human, mouse and rat genomes using real promoters sequences (black) and background sequences (white).

*** p-value<0.001 calculated by Fisher's exact test.
