# Supplementary material for: Transcription factor site dependencies in human, mouse and rat genomes
Source: BMC Bioinformatics. 2009 Oct 16;10:339. doi: 10.1186/1471-2105-10-339 (PMC2770556; doi:10.1186/1471-2105-10-339)
Supplement: Additional file 2 — Distributions of number of dependent mates in human, mouse and rat genome. File containing 3 histograms of number of dependent mates for each transcription factor in human, mouse and rat genome. [file 1471-2105-10-339-S2.PDF]

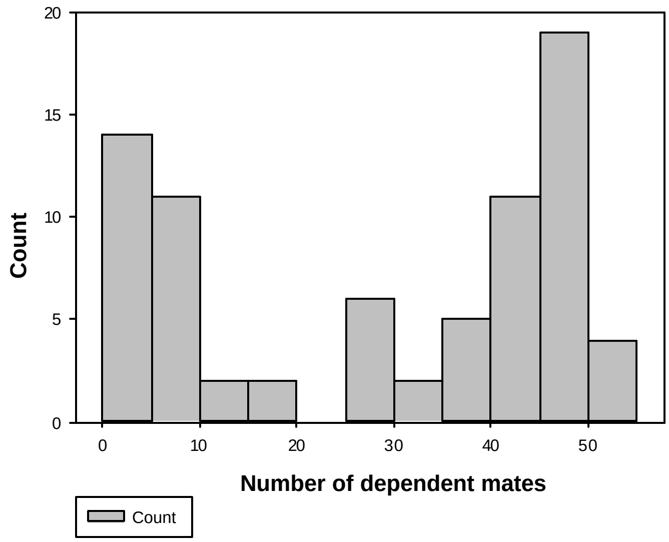

Histogram of number of dependent mates for each transcription factor in rat genome.

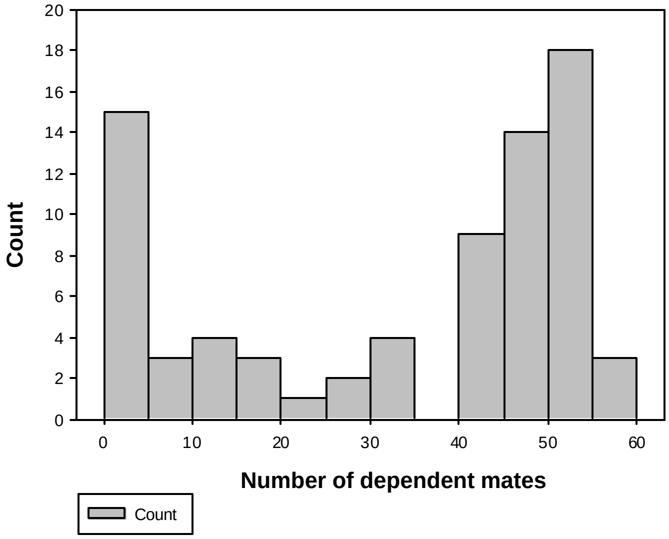

Histogram of number of dependent mates for each transcription factor in mouse genome.

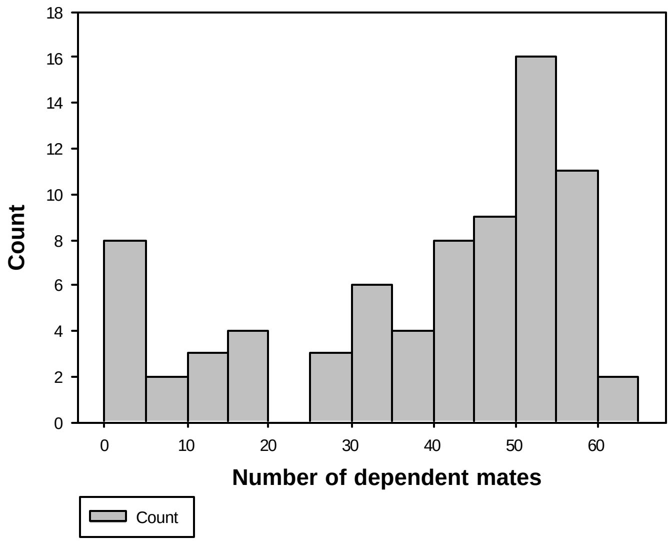

Histogram of number of dependent mates for each transcription factor in human genome.
