# Supplementary material for: Transcription factor site dependencies in human, mouse and rat genomes
Source: BMC Bioinformatics. 2009 Oct 16;10:339. doi: 10.1186/1471-2105-10-339 (PMC2770556; doi:10.1186/1471-2105-10-339)
Supplement: Additional file 3 — Distribution of dependent mates for each transcription factor in human, mouse and rat genome, including cluster information about similarity between binding sites. [file 1471-2105-10-339-S3.PDF]

The number of dependent mates for each transcription factor in human, mouse and rat genome, including cluster information (from [1]) about similarity between binding sites.

Name	TF-ID	# dep-mate-human	# dep-mate-mouse	# dep-mate-rat	Cluster (from [1])
Pparg	MA0066	0	0	0	Cluster-77
pax6	MA0069	0	0	0	Cluster-71
p53	MA0106	0	0	0	Cluster-78
Roaz	MA0116	0	0	0	-
pprag-rxra	MA0065	1	0	0	Cluster-91
hnf4a	MA0114	1	1	0	Cluster-32
nr1h2-rxra	MA0115	2	1	0	-
pax5	MA0014	3	2	0	Cluster-98
evi1	MA0029	8	6	0	Cluster-10
Srf	MA0083	6	5	1	Cluster-72
spi1	MA0080	39	0	2	Cluster-69
mzf1_1-4	MA0056	15	0	4	Cluster-85
myc-max	MA0059	16	10	4	Cluster-15
Nfya	MA0060	11	13	4	Cluster-46
gata2	MA0036	41	0	5	Cluster-9
TCF11-MafG	MA0089	33	7	5	Cluster-26
ddidt3-cebpa	MA0019	18	18	6	-
gata3	MA0037	31	0	7	Cluster-16
Spib	MA0081	14	18	7	Cluster-69
en1	MA0027	40	0	8	Cluster-8
ets1	MA0098	41	0	8	Cluster-2
zeb1	MA0103	13	13	8	-
pax2	MA0067	32	13	8	Cluster-30
yy1	MA0095	15	0	9	Cluster-94
nr3c1	MA0113	27	29	9	-
sox9	MA0077	28	18	12	Cluster-20
irf2	MA0051	32	34	13	Cluster-6
foxd3	MA0041	27	22	16	Cluster-17
Mafb	MA0117	31	26	17	-
sp1	MA0079	36	33	25	Cluster-74
mzf_1_513	MA0057	33	30	27	Cluster-99
bapx1	MA0122	47	41	28	-
rreb1	MA0073	43	44	28	Cluster-68
nkx2-5	MA0063	43	30	29	Cluster-41
esr1	MA0112	45	44	29	-
mef2a	MA0052	45	42	32	Cluster-82
T	MA0009	44	43	34	-
tlx1-nfic	MA0119	44	42	35	-
spz1	MA0111	42	42	36	-
rxra-vdr	MA0074	46	46	36	Cluster-91
nfkb1	MA0105	39	43	38	Cluster-5
Gfi	MA0038	48	47	38	Cluster-88
Sry	MA0084	52	40	40	Cluster-20

nhlh1	MA0048	38	47	41	-
nr2f1	MA0017	51	51	41	Cluster-2
hand1-tcfe2a	MA0092	48	47	42	Cluster-92
pbx1	MA0070	50	51	42	Cluster-73
elk1	MA0028	49	49	43	Cluster-2
nf-kappab	MA0061	45	48	44	Cluster-5
Mycn	MA0104	47	48	44	Cluster-15
nfil3	MA0025	51	49	44	-
Staf	MA0088	52	49	44	Cluster-81
runx1	MA0002	55	52	44	-
tal1-tcf3	MA0091	51	48	45	Cluster-7
foxd1	MA0031	50	49	45	Cluster-17
rora_2	MA0072	55	49	45	Cluster-93
Cebpa	MA0102	55	49	45	Cluster-14
Max	MA0058	50	50	45	Cluster-15
arnt-ahr	MA0006	50	51	45	Cluster-15
Rel	MA0101	51	50	46	Cluster-5
prrx2	MA0075	52	50	46	-
sox5	MA0087	55	45	47	Cluster-20
foxa2	MA0047	54	50	47	-
Gabpa	MA0062	54	52	47	-
Rela	MA0107	52	53	47	Cluster-5
Ar	MA0007	56	57	47	-
elk4	MA0076	52	52	48	Cluster-2
sox17	MA0078	54	52	48	-
foxq1	MA0040	55	52	48	-
usf1	MA0093	58	53	48	Cluster-15
tcf1	MA0046	58	54	48	Cluster-21
e2f1	MA0024	55	55	48	Cluster-35
rora_1	MA0071	59	52	50	Cluster-44
Hlf	MA0043	63	54	50	Cluster-23
Arnt	MA0004	58	55	51	Cluster-15
Creb	MA0018	61	52	53	Cluster-3

Reference:

1. Kielbasa, S.M., D. Gonze, and H. Herzel, **Measuring similarities between transcription factor binding sites.** BMC Bioinformatics, 2005. 6: p. 237.
