# Supplementary material for: Transcription factor site dependencies in human, mouse and rat genomes
Source: BMC Bioinformatics. 2009 Oct 16;10:339. doi: 10.1186/1471-2105-10-339 (PMC2770556; doi:10.1186/1471-2105-10-339)
Supplement: Additional file 4 — Distribution of GC content in the human, mouse and rat promoters. File containing 3 histograms and corresponding fitted normal distributions. [file 1471-2105-10-339-S4.PDF]

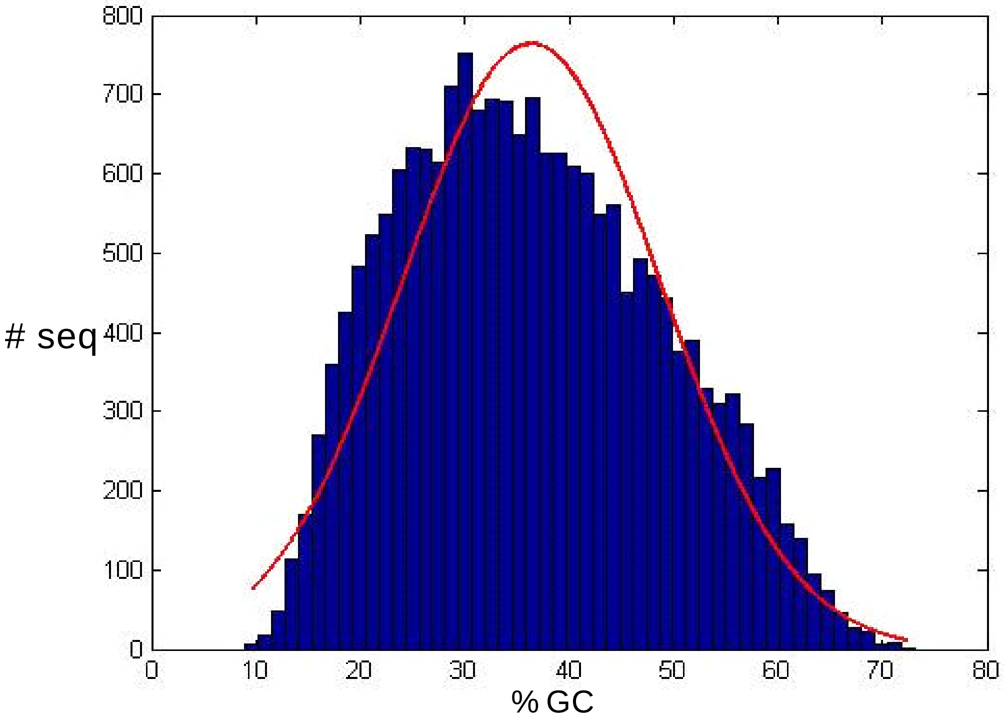

Distribution of GC content in human promoters. Red line represents fitted normal distributions (with mean 36.37 and standard deviation 12.39).

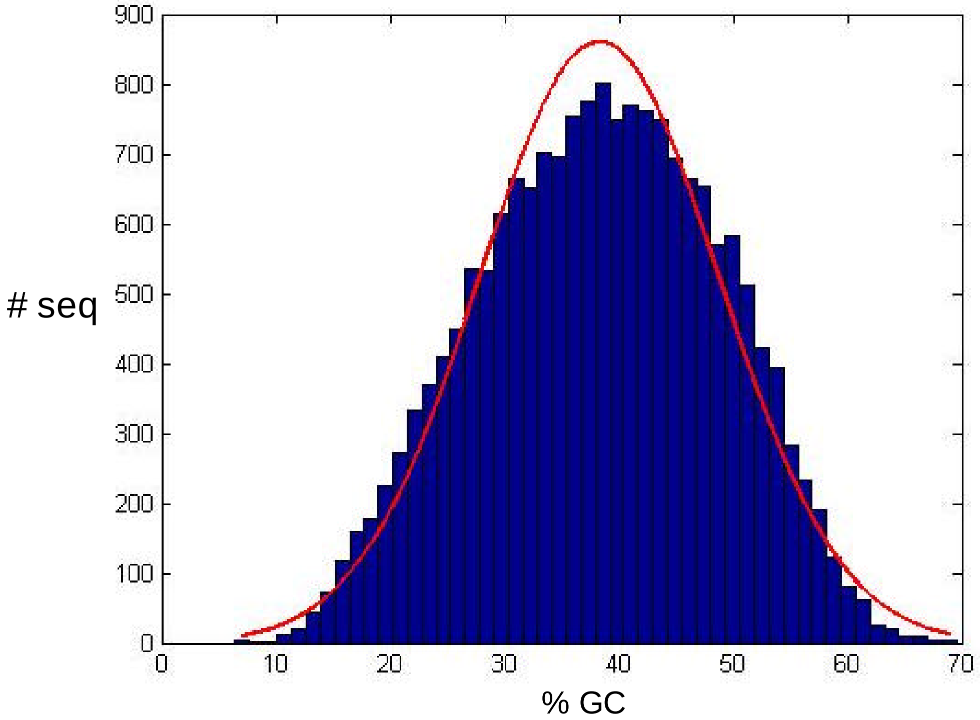

Distribution of GC content in mouse promoters. Red line represents fitted normal distributions (with mean 38.31 and standard deviation 10.52).

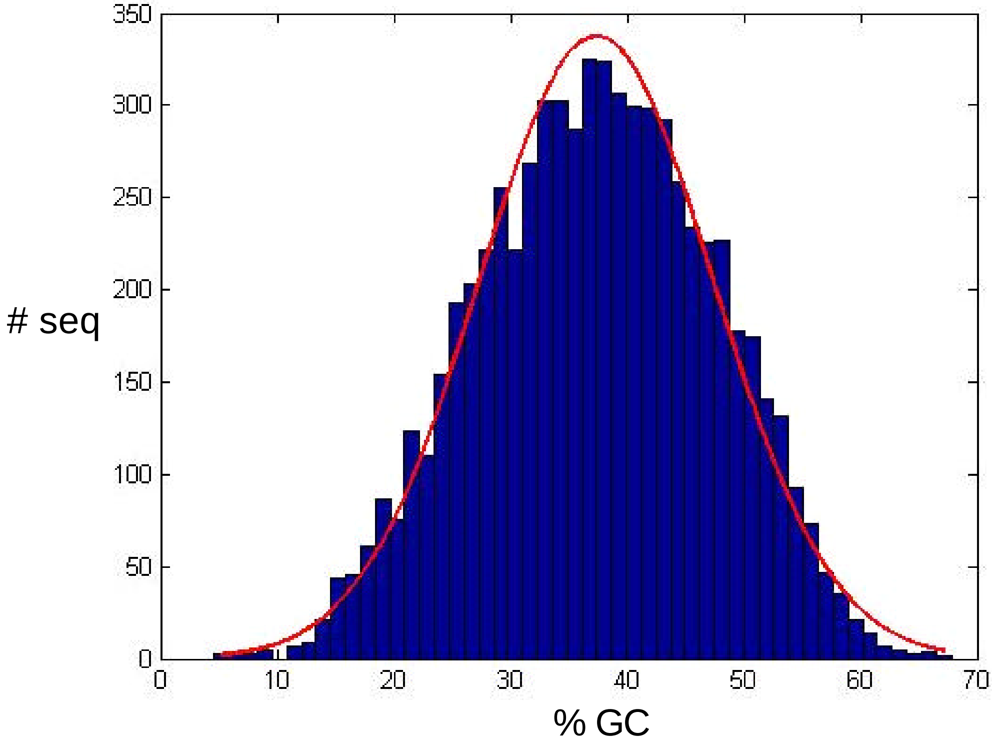

Distribution of GC content in rat promoters. Red line represents fitted normal distributions (with mean 37.38 and standard deviation 10.04).
