# Supplementary material for: Transcription factor site dependencies in human, mouse and rat genomes
Source: BMC Bioinformatics. 2009 Oct 16;10:339. doi: 10.1186/1471-2105-10-339 (PMC2770556; doi:10.1186/1471-2105-10-339)
Supplement: Additional file 5 — Scanning promoter sequences. File containing a table that represents a general form of output after scanning promoter sequences for the given combination of transcription factors A and B. [file 1471-2105-10-339-S5.PDF]

Scanning promoter sequences

General form of output after scanning promoter sequences for the given combination of transcription factors A and B.

**transcription factors promoter sequence id (%GC)**	**A**	**B**
Prom-id1 (P_{id1})%	A_1	B_1
Prom-id2 (P_{id2})%	A_2	B_2
….		
Prom-idn (P_{idn})%	A_n	B_n
