# Supplementary material for: Transcription factor site dependencies in human, mouse and rat genomes
Source: BMC Bioinformatics. 2009 Oct 16;10:339. doi: 10.1186/1471-2105-10-339 (PMC2770556; doi:10.1186/1471-2105-10-339)
Supplement: Additional file 6 — Distributions of GC content in human promoters, represented by a histogram of 50 bins. File containing 3 histograms of 50 bins each. [file 1471-2105-10-339-S6.PDF]

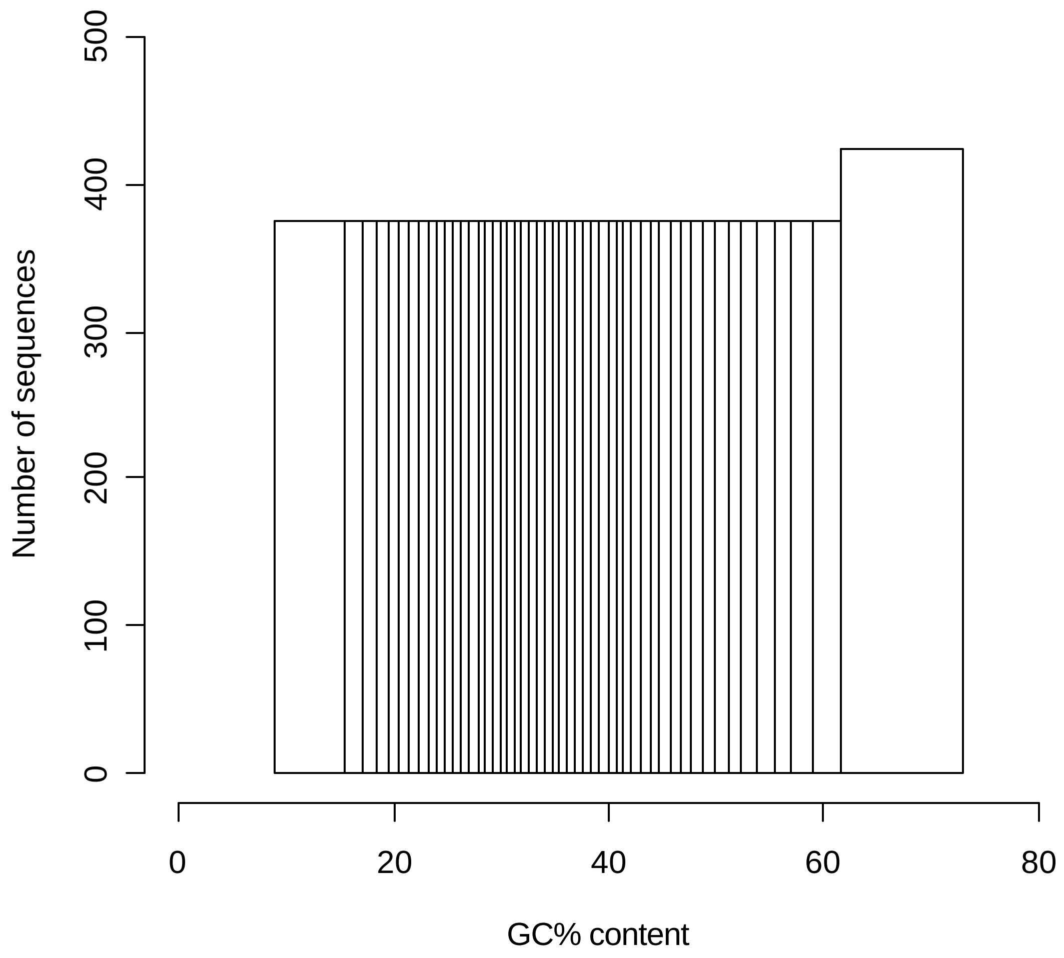

Distributions of GC content in human promoters, represented by a histogram of 50 bins each of which contains exactly 375 sequences except for the final bin that contains 424 sequences. The bin borders are given by the following array: 8.981424781, 5.48242334, 17.12593356, 18.41206602, 19.41747573, 20.44093631, 21.36474411, 22.29249012, 23.12616698, 23.98498806, 24.7291441, 25.47984645, 26.20016273, 27.00460829, 27.80918728, 28.46153846, 29.14967054, 29.84140234, 30.46171171, 31.15845539, 31.82561308, 32.55119454, 33.24845398, 33.94316855, 34.64765101, 35.33834586, 36.08159796, 36.78516229, 37.51434034, 38.29867675, 39, 39.85849057, 40.58823529, 41.41176471, 42.18403548, 43.12402698, 43.92567889, 44.80557168, 45.84569733, 46.84638861, 47.81105991, 48.85386819, 49.95495495, 51.23558484, 52.43632337, 53.92271663, 55.46651402, 57, 59.02891435, 61.63124641, 73.

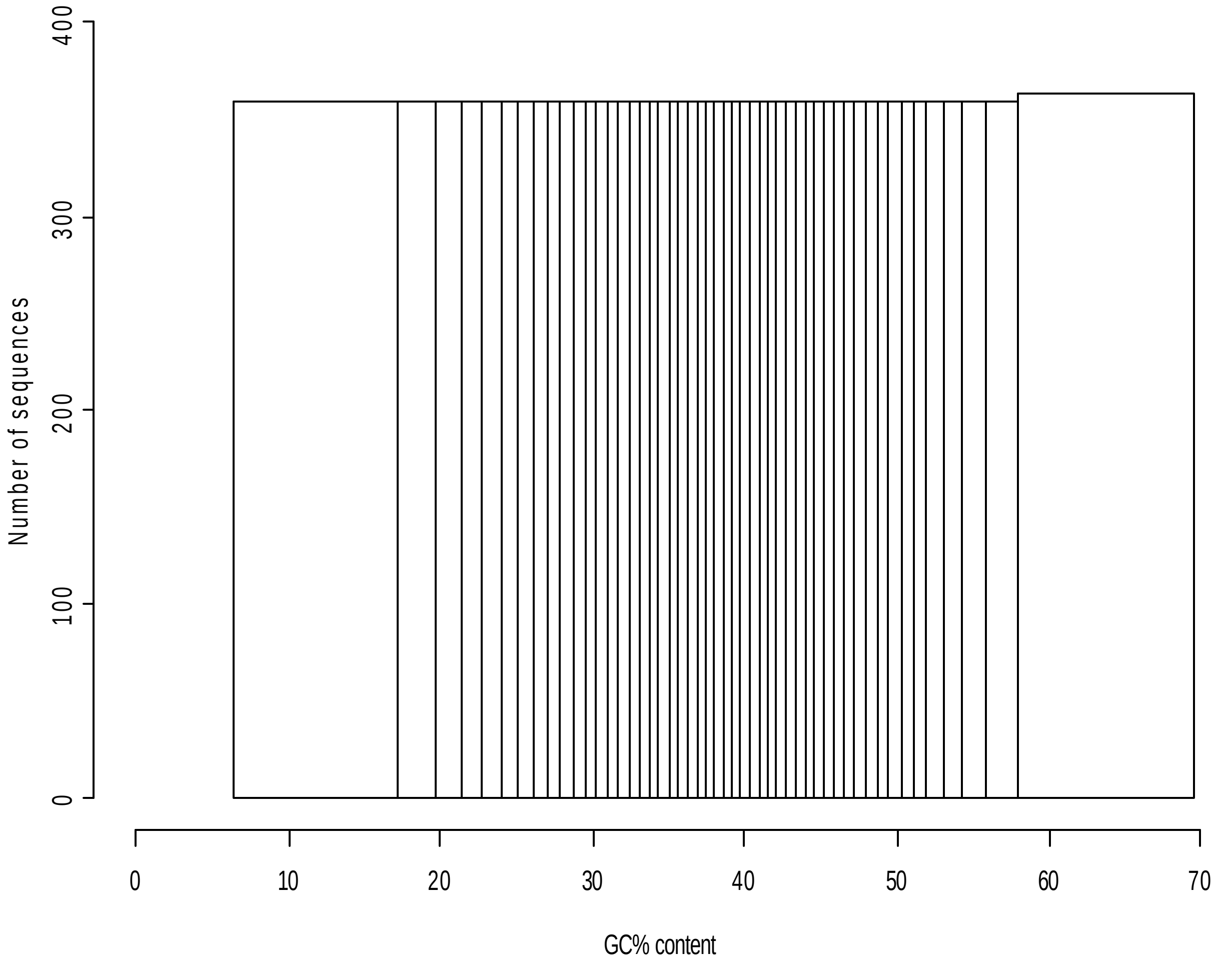

Distribution of GC content in mouse promoters, represented by a histogram made up of 50 bins each of which contains 359 promoters except for the final bin which contains 363. The bin borders are given by the following array: 6.352941176, 17.2403734, 19.63499056, 21.45005012, 22.78725459, 24.02251185, 25.10714286, 26.1328125, 27.07472775, 27.90522753, 28.77659574, 29.55508475, 30.2972561, 31.01289134, 31.6612141, 32.36404834, 33.04413429, 33.70245546, 34.35047951, 35.03348789, 35.6396217, 36.23529412, 36.83417085, 37.40388964, 38, 38.58823529, 39.13783324, 39.70588235, 40.34151547, 40.95908491, 41.55273438, 42.09115282, 42.70242393, 43.30949949, 43.92419175, 44.53870626, 45.18167457, 45.82352941, 46.50491046, 47.23360656, 47.92176039, 48.71520343, 49.47058824, 50.28735632, 51.17647059, 52, 53.11764706, 54.23529412, 55.76470588, 57.93103448, 69.55017301.

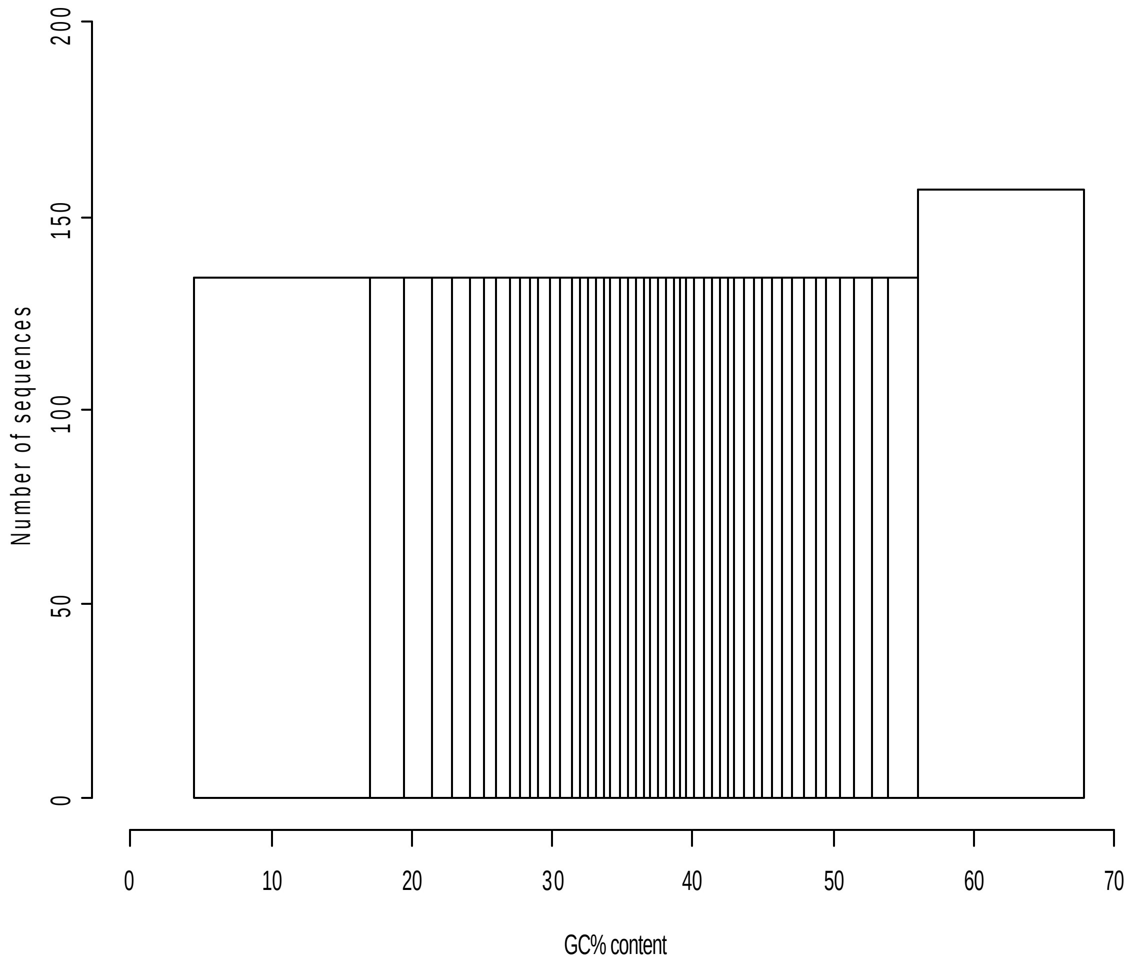

Distribution of GC content in rat promoters, represented by a histogram with a fixed number of 50 bins each of which contains 134 promoters except for the final bin which contains 157. The bin borders are given by the following array: 4.529411765, 16.98636469, 19.52821738, 21.3672391, 22.79411765, 24.12698413, 25.11578197, 25.98833441, 26.88277669, 27.65567766, 28.38883283, 29.07372401, 29.74322397, 30.60765191, 31.29147524, 31.89577718, 32.45967742, 33.05882353, 33.59240069, 34.12556054, 34.70588235, 35.3219697, 35.84715938, 36.42384106, 36.95968917, 37.49324689, 38.0010983, 38.55721393, 39.05411994, 39.59854015, 40.13909588, 40.74844075, 41.31355932, 41.94117647, 42.45951417, 43.01994302, 43.60097324, 44.26054458, 44.92273731, 45.60622914, 46.33885623, 47.05882353, 47.91785511, 48.66589327, 49.52941176, 50.50223214, 51.41176471, 52.65511459, 53.94117647, 55.92672414, 67.74916013.
