# Supplementary material for: Transcription factor site dependencies in human, mouse and rat genomes
Source: BMC Bioinformatics. 2009 Oct 16;10:339. doi: 10.1186/1471-2105-10-339 (PMC2770556; doi:10.1186/1471-2105-10-339)
Supplement: Additional file 7 — Representation of higher order dependencies between transcription factors A, B and C. File containing fully connected graph (represents full 3-order dependencies) and not fully connected graph (represents partial 3-order dependencies). [file 1471-2105-10-339-S7.PDF]

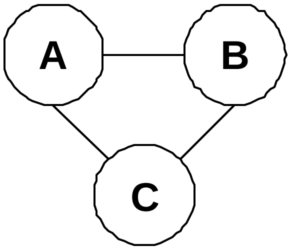

i) fully connected graph represents full 3-order dependencies between transcription factors A,B and C

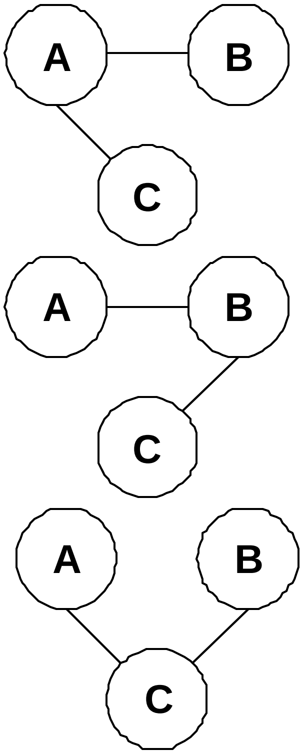

ii) Not fully connected graph represents partial 3-order dependencies between transcription factors A,B and C

Representation of higher order dependencies between transcription factors A, B and C.
